# Antibody-Based Biolayer Interferometry Platform for Rapid Detection of Neutrophil Gelatinase-Associated Lipocalin

**DOI:** 10.3390/bios15120781

**Published:** 2025-11-27

**Authors:** Somphot Saoin, Sawitree Nangola, Kannaporn Intachai, Eakkapote Prompunt, Chiraphat Kloypan, Trairak Pisitkun, Chatikorn Boonkrai

**Affiliations:** 1 Division of Clinical Immunology and Transfusion Science, Department of Medical Technology, School of Allied Health Sciences, University of Phayao, Phayao 56000, Thailand; 2 Division of Clinical Hematology and Microscopy, Department of Medical Technology, School of Allied Health Sciences, University of Phayao, Phayao 56000, Thailand; 3 Division of Clinical Microbiology and Medical Parasitology, Department of Medical Technology, School of Allied Health Sciences, University of Phayao, Phayao 56000, Thailand; 4 Department of Pathology, School of Medicine, University of Phayao, Phayao 56000, Thailand; 5 Center of Excellence in Systems Biology, Research Affairs, Faculty of Medicine, Chulalongkorn University, Bangkok 10330, Thailand

**Keywords:** biolayer interferometry, biosensor, Lipocalin-2, anti-NGAL antibody, acute kidney injury

## Abstract

Neutrophil gelatinase-associated lipocalin (NGAL) has emerged as a critical biomarker for the early diagnosis of acute kidney injury (AKI). The development of novel detection platforms that combine rapid analysis with high sensitivity is essential for improving clinical outcomes. In this study, we established an antibody-based detection system for NGAL using biolayer interferometry (BLI), a label-free optical biosensing technique that monitors real-time interference patterns generated by white light reflected from biomolecular binding events on a biosensor surface. A panel of six anti-NGAL monoclonal antibodies was generated and characterized for its binding properties, identifying candidates with high specificity for NGAL. For robust sensor functionalization, selected monoclonal antibodies were biotinylated and immobilized onto streptavidin-coated biosensor tips, establishing a stable and efficient detection interface. The optimized BLI platform demonstrated a limit of detection (LOD) of 46.1 ng/mL with wild dynamic range of 19 to 40,000 ng/mL. The platform’s accuracy was validated using human serum samples, with spike-and-recovery experiments yielding recovery rates of 96.6–104.6%. This demonstrates the capability to accurately quantify NGAL under physiologically relevant conditions with minimal matrix interference. Furthermore, the real-time kinetic measurements enabled rapid analysis, with the entire assay completed in less than half an hour. These findings establish a proof-of-concept for a BLI-based biosensor for NGAL detection, demonstrating sensitivity and specificity that show potential for clinical applications.

## 1. Introduction

Neutrophil gelatinase-associated lipocalin (NGAL) has emerged as a promising biomarker for the early detection of acute kidney injury (AKI) [1]. Conventional analyses depend on serum creatinine; unfortunately, this predictive marker is a delayed indicator and rises after kidney dysfunction [2]. NGAL is rapidly upregulated and released into the blood and urine within hours following renal tubular injury, preceding the increases in traditional markers, thereby enabling much earlier identification of kidney damage [3,4]. Early detection through NGAL measurement enables timely intervention, which is crucial for preventing further renal injury, enhancing patient outcomes, and reducing the risk of progression to chronic kidney disease or the need for renal replacement therapy [3,4,5]. This early predictive capability, along with its stability in biological fluids, has led to NGAL’s growing adoption in critical care settings for the timely identification and management of patients at risk for AKI.

Approaches for determining NGAL are now gaining importance and have been developed on various platforms. The traditional techniques include enzyme-linked immunosorbent assay (ELISA) [6], chemiluminescent immunoassays (CLIA/CMIA) [7], and fluorescence immunochromatographic test strips [8]; each has its benefits and limitations, including time-consuming processes, high costs, and difficulty of use. To overcome these limitations, novel technologies should be developed to address the drawbacks of conventional methods.

Biolayer interferometry (BLI) is a sophisticated, label-free optical biosensing technique that has emerged as a powerful analytical platform for the real-time analysis of biomolecular interactions [8]. This technology measures the interference patterns of white light reflected from a biosensor tip, where changes in the biological layer thickness, caused by molecular binding, shift the interference pattern. This allows for precise determination of the binding kinetics, affinity, and analyte concentration without requiring molecule labeling. BLI offers several advantages over traditional methods, such as surface plasmon resonance (SPR) and ELISA. This technology demonstrates high sensitivity, low sample consumption, and compatibility with complex sample matrices [9]. Furthermore, BLI is a less labor-intensive method that provides immediate details of the biomolecule binding under investigation.

To develop a rapid and sensitive method for detecting NGAL, a novel BLI-based antibody platform was established. A panel of six anti-NGAL monoclonal antibodies was produced through standard hybridoma technology, and the antibody demonstrating the most favorable binding characteristics was selected for biotinylation to achieve oriented immobilization. The biotinylated antibodies were immobilized onto streptavidin-coated sensor tips, allowing for the efficient and stable capture of NGAL from test samples. The integration of our novel monoclonal antibody with BLI technology establishes a robust analytical platform capable of rapid and precise quantification of NGAL, demonstrating significant potential for clinical diagnostic applications and biomarker research.

## 2. Materials and Methods

### 2.1. Production and Purification of Recombinant NGAL

Recombinant human NGAL protein (rhNGAL) was generated in a mammalian system according to a previously described procedure [10]. Briefly, HEK293T cells were transfected with the NGAL-expressing vector pcDNA3.1-rhNGAL/IRES/EGFP and incubated at 37 °C in 5% CO_2_. Half of the culture supernatant was harvested, and fresh medium containing DMEM supplemented with 10% FBS was added every 2 days for 14 days. The pooled supernatant was subsequently centrifuged at 2500× *g* for 30 min and then mixed with binding buffer and applied to a nickel nitrilotriacetate (Ni-NTA, Ni-Sepharose) column. All elution fractions were analyzed using sodium dodecyl sulfate–polyacrylamide gel electrophoresis (SDS-PAGE) with Coomassie blue staining and Western blotting using anti-6×His-tagged as a detector antibody. Fractions containing NGAL protein were concentrated using an Amicon Ultra-4 10 kDa centrifugal filter (Merck Millipore, Burlington, MA, USA) via centrifugation at 2500× *g* and 4 °C. The purified NGAL concentrations were measured using a Human Lipocalin-2/NGAL Duo Set ELISA kit (R&D Systems, Minneapolis, MN, USA). The produced protein was aliquoted into appropriate portions for single-use applications and stored at −70 °C to avoid repeated freeze–thaw cycles.

### 2.2. Binding Activity Testing of Monoclonal Anti-NGAL

Six selected clones of the monoclonal antibody (mAb) were tested for their binding reactivity against rhNGAL using indirect ELISA. A quantity of 10 μg/mL of 6×His-tagged rhNGAL was coated and left overnight at 4 °C in a moisture chamber; 6×His-tagged PD-L1 was used as the control protein. The coated wells were washed with washing buffer (0.05% Tween-20 in PBS) and blocked with blocking solution (1% BSA in PBS). A quantity of 10 μg/mL mAb was added to each coated well and incubated at RT for 1 h. The binding of each antibody against NGAL was detected using goat anti-mouse immunoglobulins conjugated to horseradish peroxidase (HRP). After the last washing, the TMB substrate was added, and the reaction was stopped with 1N HCl.

To test the binding activity via Western blot, the rhNGAL was separated by means of SDS-PAGE using 12% (*w*/*v*) separating gel under both reducing and non-reducing conditions. The proteins were transferred to PVDF membranes and individually cut for incubation with each mAb. Each membrane strip was further incubated with 2% BSA in PBS as a blocking buffer. Each strip was incubated with the six monoclonal antibodies and detected using HRP-conjugated goat anti-mouse immunoglobulins (Bio-Rad, Hercules, CA, USA). The reactive bands were visualized with a TMB membrane peroxidase substrate system (KPL, Gaithersburg, MD, USA).

### 2.3. Biotinylation of Monoclonal Antibody and Binding Activity Testing

Anti-NGAL mAbs or isotype-matched control mAbs were biotinylated using an EZ-Link™ Sulfo-NHS-LC-Biotin kit (Thermo Fisher Scientific, Waltham, MA, USA) according to the manufacturer’s instructions. Briefly, purified anti-NGAL mAb, clone 28H5, at 10 mM was mixed with a 20-fold molar excess of Sulfo-NHS-Biotin solution and incubated at 25 °C for 30 min. Excess by-products and reagents were eliminated by applying the mixture to a pre-equilibrated Vivaspin column with polyethersulfone (PES) membranes at a 50 kDa molecular weight cut-off (Sartorius, Göttingen, Germany). The concentration of biotinylated antibody was measured using Bradford reagent (Bio-Rad, Hercules, CA, USA). The biotinylation efficiency of the proteins was qualitatively evaluated using Western blotting with HRP-conjugated streptavidin (SA-HRP) as the detector. In addition, the biotinylated mAbs were evaluated for their binding activity using an indirect ELISA under the same conditions mentioned above, but with SA-HRP as the detector.

### 2.4. Antibody Optimization for Biosensor

The streptavidin (SA) biosensor was hydrated in the sample diluent for 10 min before the binding process began. The biotinylated antibody was diluted in the fresh sample diluent (0.2% bovine serum albumin (BSA), 0.01% Tween-20 in phosphate-buffered saline (PBS), pH 7.2) at concentrations of 5, 10, 50, and 100 μg/mL. The different concentrations of biotinylated anti-NGAL were further immobilized on the hydrated tip for 120 s, referred to as the loading step. Then, the saturated antibody on the biosensor tip was washed with the sample diluent for 30 s to remove excess antibodies, a step referred to as dissociation. The binding signal of the mAb was monitored in real time using a biolayer interferometry (BLI) biosensor (FortéBio, Menlo Park, CA, USA).

### 2.5. Biosensor Platform for NGAL Detection

All interaction analyses were performed in the sample diluent (0.2% BSA, 0.01% Tween-20 in PBS, pH 7.2). Initially, the SA biosensor tip was hydrated for 10 min. Biotinylated anti-NGAL monoclonal antibody (5 μg/mL) was further immobilized on the hydrated tip for 120 s (loading step). The saturated biosensors were washed in sample diluent for 30 s and then transferred to tubes containing various concentrations (19, 312, 1250, and 10,000 ng/mL) of rhNGAL diluted in sample diluent for 120 s (association step). Finally, the biosensors were washed with the sample diluent for 120 s (dissociation step). Binding signals were obtained using a BLItz biolayer interferometry biosensor (FortéBio, Menlo Park, CA, USA). The kinetic parameters (k_a_ and k_d_) and the equilibrium dissociation constant (KD) binding signal were analyzed using the BLItz Pro 1.3 program.

### 2.6. Construction of Calibration Curve

Biotinylated monoclonal antibody at a concentration of 5 μg/mL was immobilized onto the sensor tip for 120 s. Subsequently, serially diluted rhNGAL in sample diluent, ranging from 19 to 40,000 ng/mL, was applied to the sensor for 120 s during the association phase, followed by immersion in diluent for 120 s during the dissociation phase. Binding signals were acquired using a BLItz biolayer interferometry biosensor (FortéBio, Menlo Park, CA, USA). Triplicate samples were examined for each concentration. Delta binding signals were calculated and plotted according to the methodology described in the Section 2.9. The limit of detection (LOD) was determined using PD-L1 as a negative control protein to calculate the assay’s sensitivity.

### 2.7. Serum Samples

Normal human serum was purchased from Merck Millipore (EMD Millipore, Temecula, CA, USA; Cat no. S1-100ML; Lot no. 4257091). The clinical samples from patients were leftover samples from the clinical laboratory at Nan Hospital. The protocol for the collection and preparation of human serum was performed in accordance with the Declaration of Helsinki and received approval from the Institutional Review Board (IRB) of the University of Phayao, Thailand (Approval number: HREC-UP-HSST 1.1/034/68), and Nan Hospital, Thailand (Approval number: Nan HOS. REC No. 020/2568).

### 2.8. NGAL Quantification in Serum

For the quantification of NGAL, rhNGAL protein was spiked into normal human serum (Merck Millipore, USA). To mitigate matrix effects, the serum was diluted at ratios ranging from 1:50 to 1:100, selected through preliminary optimization. All experimental parameters, including the BLItz biosensor protocol and duration, were consistent with those used for calibration curve generation. Delta binding signals were determined and then used to calculate NGAL levels by referencing the calibration curve.

### 2.9. Data Analysis

The biosensor binding responses were quantified as delta binding signals, calculated by subtracting the binding signal at time point 180 min (starting point (SP) at minute 180; SP_180_) from that at the end point (EP) at time point 299.8 min (EP_299.8_), as expressed in the following equation:

Delta binding signal = EP_299.8_ − SP_180_

For the calibration curve measurement, the limit of detection (LOD) was determined using the following equation:

LOD signal = Average of the delta binding signal of control + 3SD of control

The calculated LOD signals (estimated from four replicates of the control signal) were substituted as the Y-value in the calibration curve equation, and the corresponding X-value was then determined to establish the LOD.

## 3. Results

### 3.1. Specificity of Monoclonal Antibodies Against NGAL

**Figure 1:**
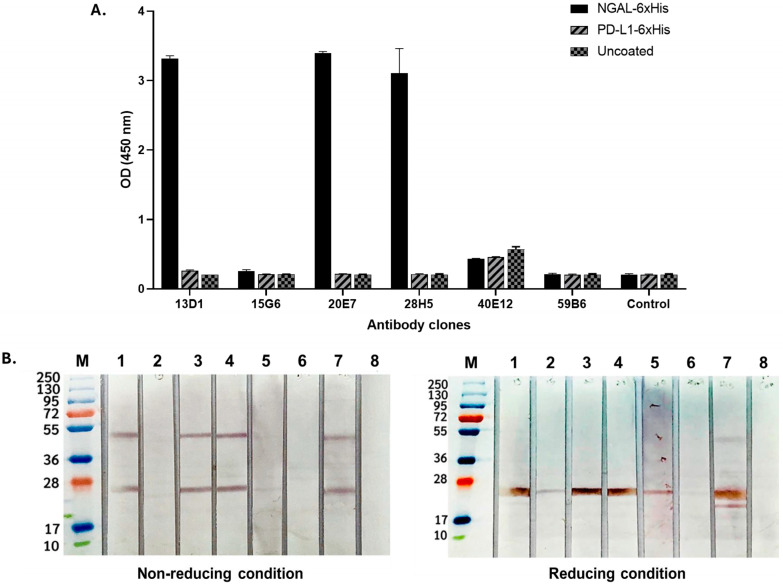
Evaluation of monoclonal antibodies for specificity against NGAL. (**A**) Detecting the binding activity of all monoclonal antibody clones via indirect ELISA. The rhNGAL was coated, followed by the addition of each monoclonal antibody clone (13D1, 15G6, 20E7, 28H5, 40E12, and 59B6). The isotyped antibody was used as a control. The immune complex was detected using goat anti-mouse–HRP conjugate. (**B**) Analysis of antibody characteristics in the presence of antigens under non-reducing (**left**) and reducing (**right**) conditions. This was conducted by means of Western blotting, which involved separating the rhNGAL protein into strips and subsequently exposing each strip to individual antibody clones. The banding was detected using monoclonal anti-His tag antibodies followed by anti-mouse–HRP conjugate. Lane M: protein ladder; Lane 1: 13D1; Lane 2: 15G6; Lane 3: 20E7; Lane 4: 28H5; Lane 5: 40E12; Lane 6: 59B6; Lane 7: anti-His tagged; Lane 8: conjugated control.

Monoclonal antibodies targeting the NGAL antigen were evaluated for their ability to bind exclusively to NGAL, with no cross-reactivity with His-tagged peptides or the irrelevant protein PD-L1. The results demonstrated that the antibody clones 13D1, 20E7, and 28H5 exhibited high binding reactivity with His-tagged recombinant human NGAL (rhNGAL) and showed no cross-reactivity with 6xHis-tagged PD-L1 (Figure 1A). This indicated specificity for the NGAL protein, rather than binding to the His tag. Notably, Western blot analysis under both reducing and non-reducing conditions of the NGAL protein revealed that these three antibody clones could recognize the NGAL protein at approximately 25 and 45 kDa under non-reducing conditions (Figure 1B, left) and at 25 kDa under reducing conditions (Figure 1B, right). These observations indicate that these three NGAL-specific antibody clones are capable of recognizing the antigen in both its native and linear conformations. Furthermore, the binding suggests recognition of NGAL in both its monomeric and homodimeric forms.

### 3.2. Characterization of Biotinylated Anti-NGAL Monoclonal Antibody

**Figure 2:**
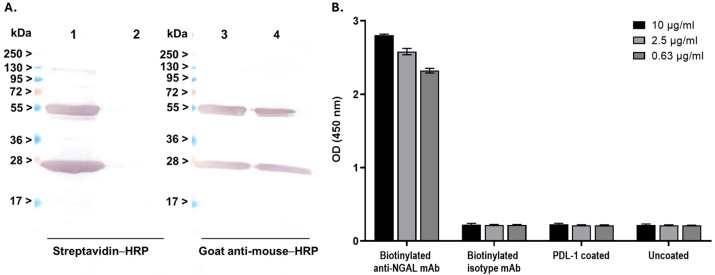
Characterization of the biotinylated anti-NGAL antibody: (**A**) An assessment of biotinylation efficiency. Biotinylated anti-NGAL antibody was subjected to Western blot analysis and detected using streptavidin-conjugated horseradish peroxidase (SA-HRP) (**left** panel) and goat anti-mouse–HRP (**right** panel). Lanes 1 and 3: biotinylated anti-NGAL antibody; Lanes 2 and 4: wild-type anti-NGAL antibody (non-biotinylated). (**B**) Evaluation of binding activity after biotinylation. The ELISA procedure was performed in accordance with the previous experiment, with SA-HRP utilized as the detector.

The anti-NGAL antibody clone 28H5 was selected from the three clones that demonstrated high binding activity with NGAL for labeling with biotin. Following the biotinylation and purification processes, the labeled antibody was evaluated via Western blot analysis with SA-HRP as the detector. The results revealed distinct bands at approximately 25 kDa and 50 kDa, which are consistent with the expected molecular weights of biotinylated light and heavy chains, respectively (Figure 2A, left; Lane 1). These bands were notably absent in the unlabeled antibody (Figure 2A, left; Lane 2). Additionally, the antibody’s identity was confirmed by using goat anti-mouse–HRP for detection (Figure 2A, right; Lanes 3 and 4). The biotinylated antibody was further assessed through binding assays. It was demonstrated that the biotinylated antibody maintained its binding activity with the NGAL target across all concentrations (0.63, 2.5, and 10 μg/mL), but not with the isotype control (Figure 2B). Taken together, these results show that the monoclonal antibody can be effectively biotinylated while preserving its target binding affinity for NGAL.

### 3.3. Optimization for Antibody Capture

**Figure 3:**
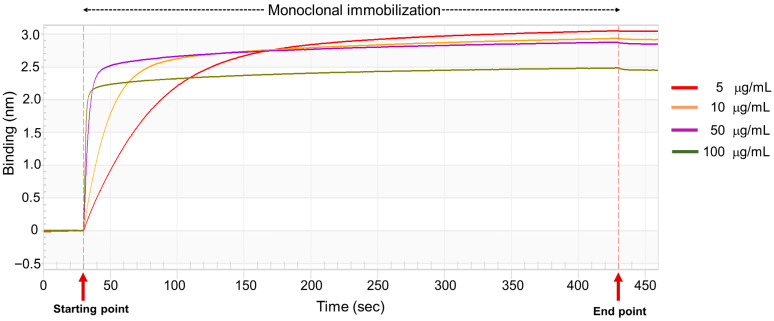
Optimization of biotinylated anti-NGAL antibody on the streptavidin sensor. The biotinylated mAb, at concentrations ranging from 5 to 100 μg/mL, was immobilized on the streptavidin sensor tip after an initial baseline measurement with the sample diluent. The binding signals were monitored using the BLItz system.

To develop a biosensor-based assay for NGAL detection, the optimal concentration of the capture antibody was initially determined using streptavidin (SA) biosensor tips. The hydrated tips were dipped into various concentrations of biotinylated antibody. Samples were tested at each mAb concentration (5, 10, 50, and 100 μg/mL). The binding signal onto the sensor was monitored in real time by using a biolayer interferometry biosensor. As shown in Figure 3, the biotinylated mAb demonstrated a consistent increase in binding signal, reaching the saturation point within 400 s during the immobilization step. The binding signal remained stable in the dissociation step, indicating steady immobilization of the biotinylated mAb on the SA biosensor tip. Comparable saturation binding responses were observed at 5, 10, and 50 μg/mL in the immobilized step, whereas a notably lower signal was detected at 100 μg/mL. Based on these results, the optimal concentration for further experiments was found to be within the range of 5–50 μg/mL.

### 3.4. BLI Platform for Detecting NGAL

**Figure 4:**
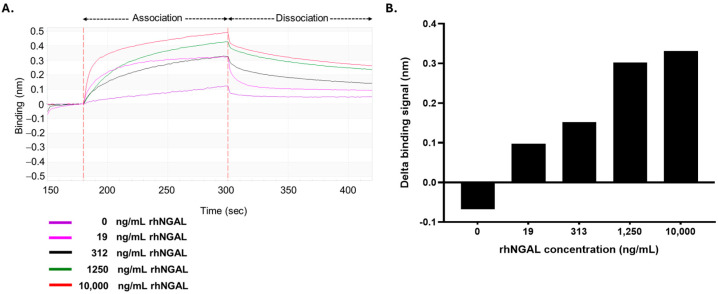
Detection of NGAL by the biosensor platform: (**A**) Sensorgrams of the binding of biotinylated mAb to NGAL. The optimal biotinylated anti-NGAL antibody was immobilized on the SA tip. The coated SA tip was subsequently tested on samples spiked with NGAL to various levels (19, 312, 1250, and 10,000 ng/mL). (**B**) The binding signals of the antibody-based biosensor systems. The delta binding signals were calculated by subtracting the signal at the starting point from that at the end point.

The performance of the model biosensor assay was evaluated using rhNGAL protein at concentrations ranging from 0 to 10,000 ng/mL. The biosensor exhibited a significantly detectable signal at levels of 313 ng/mL (R^2^ = 0.906) and higher, with a low measurable response at lower concentrations of 19 ng/mL (0.76 nM) (R^2^ = 0.763) (Figure 4). These results revealed that a BLI detection platform utilizing biotinylated anti-NGAL mAb is capable of detecting NGAL within physiologically relevant concentration ranges.

### 3.5. Calibration Curve and Sensitivity

**Figure 5:**
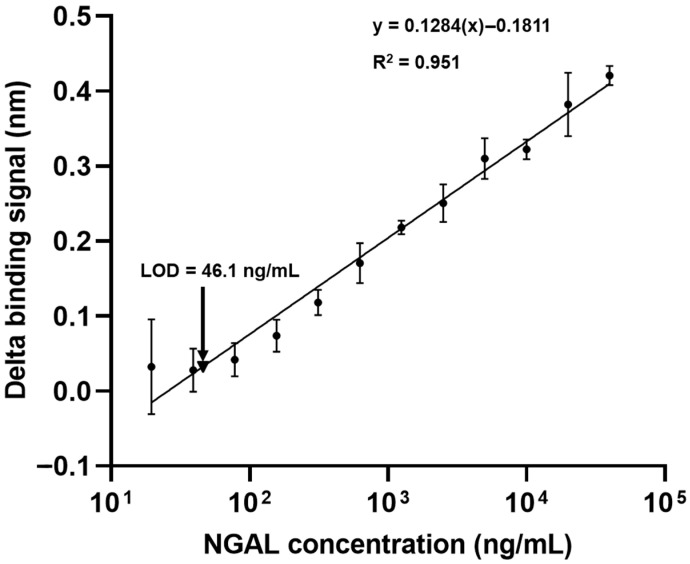
Construction of a calibration curve for NGAL quantification. Various concentrations (19–40,000 ng/mL) of NGAL were serially tested with the biotinylated mAb-coated sensor. The binding signals were measured using a BLItz biolayer interferometry biosensor. The delta binding signals of the anti-NGAL antibody on the sensors were plotted and are presented as the mean ± SD of three independent experiments. The R-squared value and equation are illustrated. The limit of detection (LOD) was calculated against the control protein. Data are the mean ± SD from three independent experiments; one sensor was tested with one concentration of NGAL.

To evaluate the immunosensor’s suitability for quantitative assays, a calibration curve was constructed using rhNGAL at concentrations ranging from 19 to 40,000 ng/mL. The delta binding signal demonstrated a linear relationship with the NGAL concentration across the studied range (R^2^ = 0.951) (Figure 5). The limit of detection (LOD) for NGAL was established by incorporating PD-L1 protein as a negative control (Figure 1A), allowing for an accurate background signal assessment for LOD calculation. Based on the calculated delta binding signal, the LOD was determined to be 46.1 ng/mL. This sensitivity is particularly relevant given that normal human serum typically presents NGAL levels below 100 ng/mL [11]. In contrast, the NGAL levels in patient serum samples showed a range from 26.8 to 1808 ng/mL [12], indicating that our immunosensor possesses sufficient sensitivity to detect NGAL within clinically relevant ranges.

### 3.6. Quantitative Assay for NGAL in Human Serum

To assess matrix effects on NGAL quantification in human serum, we compared the sensor responses obtained in serially diluted serum against those in the original assay diluent. Following antibody immobilization, the biosensors were exposed to normal human serum across a dilution series to evaluate their signal behavior during the association and dissociation phases. As shown in Figure S1, the sensorgrams for the 1:50 and 1:100 serum dilutions were comparable to those for the original diluent. In contrast, undiluted serum and 1:2, 1:5, and 1:10 dilutions demonstrated significant interference with binding signals during both the association and dissociation phases. Consequently, the optimal serum dilution range was determined to be 1:50 to 1:100. To demonstrate its analytical applicability, the BLI-based immunosensor was used to quantify NGAL in spiked human serum prepared under these optimized dilution conditions (Table 1). The recovery studies demonstrated values ranging from 96.6% to 104.6%, with a relative standard deviation (RSD) of 4.2%, well below the 5.0% threshold. These results confirm that the developed immunosensor exhibits sufficient accuracy and precision for the determination of NGAL in human serum.

## 4. Discussion

The development of methods for early biomarker detection has the potential to facilitate urgent therapy for AKI patients. Biosensors based on biolayer interferometry represent a significant advancement for label-free and real-time analysis [8,13]. Biolayer interferometry, as employed in the BLItz system, utilizes white light illumination directed toward the biosensor surface and captures the reflected light signal. The interference patterns generated by white light reflection from two distinct surfaces—an internal reference layer and a molecular binding layer at the biosensor tip—are subsequently analyzed. Molecular binding events at the biosensor tip induce alterations in the interference pattern, which are converted to quantifiable binding signals through real-time monitoring. These binding signals demonstrate correlations with both the concentration and molecular size of the target analytes. Herein, we employed this technology to detect NGAL, thereby improving the performance of the detection method.

Our study demonstrates that the developed BLI-based NGAL biosensor offers significant advantages for clinical application. The method exhibits exceptional speed, delivering quantitative results in approximately 17.5 min in contrast to the several hours typically required for conventional ELISA. The biosensor achieves high sensitivity with a limit of detection of 46.1 ng/mL with a wide dynamic range (19 to 40,000 ng/mL, Figure 5). Although a definitive cut-off for serum NGAL has not been established, prior research indicates that concentrations in patients frequently exceeded 100 ng/mL [12,14,15]. Therefore, it is suitable for early detection across the full spectrum of NGAL levels, from baseline to severe acute kidney injury. Additionally, the BLI platform requires only a minimal sample volume (4 µL), conserving precious clinical samples.

The biosensor’s performance in real biological samples demonstrates both robust capabilities and areas requiring further optimization. Matrix effect studies using normal human serum confirmed that optimal dilutions in the range of 1:50 to 1:100 effectively alleviated matrix interference while preserving antibody kinetics and maintaining signal stability (Figure S1). When used to analyze 80 clinical samples from kidney disease patients, which were previously quantified by means of ELISA (with a maximum NGAL concentration of 432.5 ng/mL), the biosensor encountered quantification difficulties for many samples after dilution, as the NGAL concentrations fell below the limit of detection due to the dilution effect (Figure 5). This highlights a specific limitation regarding dynamic range compatibility when testing samples with lower NGAL levels at higher dilution factors. However, subsequent NGAL spiking experiments in normal serum at optimized dilutions demonstrated the biosensor’s accuracy and precision in quantifying NGAL in relevant matrices, with acceptable recovery rates (Table 1). The biosensor exhibits robust reproducibility and reliability through meticulous control of matrix consistency, stable biotin–streptavidin-immobilized capture antibodies providing consistent active binding sites across multiple regeneration cycles, and streptavidin’s low non-specific binding ensuring high specificity while minimizing interference. These findings, combined with the high sensitivity and rapid turnaround time, underscore the biosensor’s potential for clinical application despite the identified limitation in detecting very low NGAL concentrations.

Biotinylation of the monoclonal antibody is a powerful immobilization strategy on streptavidin-coated biosensors, primarily ensuring exceptional assay stability due to the biotin–streptavidin interaction’s high affinity (KD~10^−14^ M) [16]. This robust, virtually irreversible bond prevents antibody leaching and maintains a stable capture surface throughout assay cycles, crucial for sensor longevity and reproducibility. However, cautious biotinylation is essential, as suboptimal placement or excessive biotinylation could potentially introduce steric hindrance, reducing the apparent binding kinetics (ka) or maximum binding capacity (Rmax) of NGAL to the antibody by affecting its accessibility or orientation. Streptavidin’s utility in biosensing stems from its tetravalency (four biotin binding sites), which enhances its immobilization density and avidity, and its inherently low non-specific binding, minimizing matrix interference and ensuring high signal-to-noise ratios; all of these contribute to a reliable and sensitive NGAL detection platform [17].

The distinction of molecular forms of NGAL represents a critical advancement in the biomarker approach, with significant clinical implications [18]. Identifying the different sources of NGAL will also help in understanding the timing of different pathophysiological events during the AKI continuum. NGAL is produced in various molecular forms in neutrophils [19], plasma [20], and urine [21]. All forms include a monomeric NGAL (25 kDa), a dimeric NGAL (45 kDa), and a heterodimer (135 kDa), covalently conjugated with gelatinase. The NGAL monomer predominantly originates from renal tubular epithelial cells and is strongly associated with AKI, making it a sensitive and early marker for kidney damage. In contrast, the dimeric form is primarily released from activated neutrophils and is more frequently observed in conditions involving urinary tract infections (UTIs) [22,23].

The clinical performance of the NGAL assay in detecting different forms of NGAL depends on the antibody configuration [24]. Current immunoassays often fail to discriminate between these forms, leading to potential misinterpretation of clinical data and suboptimal diagnostic accuracy. Likewise, in this study, our monoclonal antibodies did not identify the monomeric and dimeric forms, which is a critical gap in current diagnostic methodologies. However, Cai and co-workers studied the effect of antibody configuration on the performance of an assay and revealed that the epitope specificities used in the ELISA paralleled differences in the antibodies used to identify the different forms of urine NGAL [21]. Martensson and colleagues showed that a combination of different specific epitopes could detect NGAL in each form. Two ELISAs were developed, and demonstrated monomer-specific assays are expected to reduce any confounding effect of neutrophil involvement during bacteriuria or sepsis [25]. Nickolas and colleagues found a noticeable correlation between urine NGAL concentrations measured via immunoblot and via chemiluminescent microparticle immunoassay. Their study demonstrated that the monomeric form of urinary NGAL quantified by means of immunoblot was identical to that quantified via the standardized clinical platform, confirming that monomeric urine NGAL is the form related to AKI [26]. Furthermore, the ratio of monomeric to dimeric NGAL may serve as an additional diagnostic parameter, providing enhanced specificity for different pathological states and improving patient stratification in clinical settings [27].

In recent years, other biosensor-based approaches, particularly electrochemical and nanomaterial-based biosensors, have emerged as superior methods due to their remarkable sensitivity, lower detection limits, and rapid turnaround time [28,29,30]. For example, dual-signal electrochemical immunosensors and graphene-based or metal nanoparticle-enhanced sensors are reported to have limits of detection in the picograms-per-milliliter range, outperforming conventional assays in terms of analytical performance and enabling earlier and more precise clinical diagnoses of NGAL-related diseases [28,30]. Ongoing research continues to refine these biosensor systems, emphasizing their promise as next-generation tools for clinical NGAL monitoring [31].

## 5. Conclusions

In summary, we successfully developed an antibody-based biolayer interferometry system for quantitative NGAL measurement. Our findings demonstrate that this method provides highly sensitive detection with a limit of detection in the nanograms-per-milliliter range, meeting the requirements for clinical application in acute kidney injury diagnosis. To enhance the proposed system’s clinical utility, our next steps will focus on developing a multiplexed platform capable of simultaneously detecting other critical kidney injury biomarkers, such as KIM-1 and Cystatin C, alongside NGAL. This multi-marker approach promises to significantly improve the diagnostic precision and prognostic power for AKI, moving beyond the limitations of single-marker analysis. Furthermore, the high sensitivity and efficiency demonstrated by our developed BLI biosensor make it particularly well-suited for integration into point-of-care testing (POCT) platforms, where rapid turnaround time and ease of use are paramount. To further enhance the detection sensitivity for small-molecular-weight proteins like NGAL, we propose implementing signal amplification strategies. These include employing a secondary antibody that binds to the captured NGAL, creating a sandwich immunoassay format, or utilizing gelatinase as a signal-enhancing molecule. Since gelatinase possesses a larger molecular mass, its binding would generate substantially stronger binding signals, effectively addressing the inherent challenge of detecting small, low-abundance proteins with high precision.

## Figures and Tables

**Table 1:** Assessment of NGAL in spiked serum samples.

Spiked Concentration (ng/mL)	Measured Concentration (ng/mL)	Recovery (%)
100	98.4 ± 22.9	98.4
2000	2091.2 ± 66.9	104.6
20,000	19,320.7 ± 991.0	96.6

## Data Availability

The data supporting this study’s findings are available from the corresponding author upon reasonable request.

## Supplementary Materials

The following supporting information can be downloaded at: https://www.mdpi.com/article/10.3390/bios15120781/s1, Figure S1: Assessment of the diluent effect. Following immobilization of anti-NGAL monoclonal antibody on the sensor surface, the biosensor was exposed to normal human serum at various dilution ratios ranging from undiluted to 1:100.

## Abbreviations

The following abbreviations are used in this manuscript:

AKI	Acute kidney injury
NGAL	Neutrophil gelatinase-associated lipocalin
rhNGAL	Recombinant human neutrophil gelatinase-associated lipocalin
BLI	Biolayer interferometry
mAbs	Monoclonal antibodies
SPR	Surface plasmon resonance
ELISA	Enzyme-linked immunosorbent assay
SDS-PAGE	Sodium dodecyl sulfate–polyacrylamide gel electrophoresis
kDa	Kilodalton
BSA	Bovine serum albumin
PBS	Phosphate-buffered saline
HRP	Horseradish peroxidase
TMB	3,3′,5,5′-Tetramethylbenzidine (TMB)
SP	Starting point
EP	Endpoint
PD-L1	Programmed death-ligand 1
SA	Streptavidin
UTIs	Urinary tract infections
