# Supplementary material for: Antibody-Based Biolayer Interferometry Platform for Rapid Detection of Neutrophil Gelatinase-Associated Lipocalin
Source: Biosensors (Basel). 2025 Nov 27;15(12):781. doi: 10.3390/bios15120781 (PMC12730675; doi:10.3390/bios15120781)
Supplement: Supplementary file 1 [file biosensors-15-00781-s001.zip › biosensors-3924506-supplementary.pdf]

biosensors

Supplementary materials

Antibody-Based Biolayer Interferometry Platform for Rapid Detection of Neutrophil Gelatinase-Associated Lipocalin

Somphot Saoin [1,*], Sawitree Nangola [1], Kannaporn Intachai [2], Eakkapote Prompunt [3], Chiraphat Kloypan [4], Trairak Pisitkun [5] and Chatikorn Boonkrai [5,*]

[1] Division of Clinical Immunology and Transfusion Science, Department of Medical Technology, School of Allied Health Sciences, University of Phayao, Phayao 56000; Thailand
[2] Division of Clinical Hematology and Microscopy, Department of Medical Technology, School of Allied Health Sciences, University of Phayao, Phayao 56000, Thailand
[3] Division of Clinical Microbiology and Medical Parasitology, Department of Medical Technology, School of Allied Health Sciences, University of Phayao, Phayao 56000 Thailand
[4] Department of Pathology, School of Medicine, University of Phayao, Phayao 56000, Thailand
[5] Center of Excellence in Systems Biology, Research Affairs, Faculty of Medicine, Chulalongkorn University, Bangkok 10330, Thailand
* Correspondence: somphot.sa@up.ac.th (S.S.); ctkorn@gmail.com (C.B.)

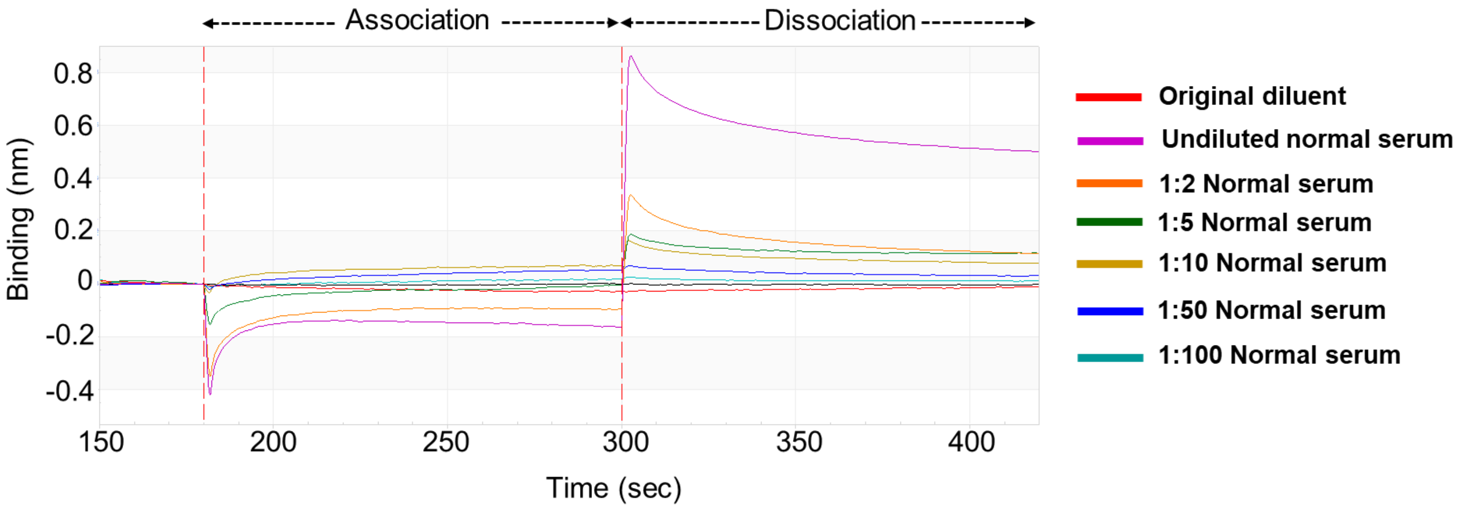

Figure S1. Assessment of the diluent effect. Following immobilization of anti-NGAL monoclonal antibody on the sensor surface, the biosensor was exposed to normal human serum at various dilution ratios ranging from undiluted to 1:100.
